# Variability of DKA Management Among Pediatric Emergency Room and Critical Care Providers: A Call for More Evidence-Based and Cost-Effective Care?

**DOI:** 10.4274/jcrpe.1434

**Published:** 2014-09-05

**Authors:** Matthew G Clark, Abdallah Dalabih

**Affiliations:** 1 Missouri-Columbia University, Columbia, MO

**Keywords:** Pediatric DKA, management, protocols, guidelines, emergency department, critical care unit

## Abstract

Management protocols have been shown to be effective in the pediatric emergency medicine (PEM) and pediatric critical care (PCC) settings. Treatment protocols define clear goals which are achieved with consistency in implementation. Over the last decade, many new recommendations have been proposed on managing diabetic ketoacidosis (DKA). Although no perfect set of guidelines exist, many institutions are developing DKA treatment protocols. We sought to determine the variability between institutions in implementation of these protocols.

## INTRODUCTION

Management protocols based on consensus guidelines, by defining clear goals to be achieved with consistency in implementation, are effective in pediatric emergency medicine (PEM) and pediatric critical care (PCC) settings. We sought to determine the extent to which providers follow current guidelines (1,2) for management of diabetic ketoacidosis (DKA) in the pediatric population and also to assess the opinions of the providers on specific treatment endpoints and on the acceptable ranges and utilization of laboratory resources.

## METHODS

We administered an e-mail-based questionnaire to PCC and PEM providers through fellowship directors in the United States. This anonymous survey consisted of 18 questions relating to the treatment of DKA. The study questionnaire was approved by the University of Missouri Institutional Review Board.

## RESULTS

We received 192 responses (74% PCC, 26% PEM providers). Eighty-nine percent of the respondents indicated having a DKA protocol at their institution. The providers’ opinions varied in defining endpoints in the resolution of DKA. The answers were: closed anion gap (46%), normal pH (26%), normal bicarbonate (34%), absence of ketones (17%) and base deficit correction (9%). For respondents who chose serum bicarbonate as the most important indicator of DKA resolution, the acceptable level varied from 12 to 20 mmol/L with 43% of these participants requiring a level of 20 mmol/L or higher. Sixty-seven percent perceived no clinical advantage in use of serum ketone measurement. Opinions on management of an insulin pump varied from 60% of respondents who advocated to always remove the pump, 5% advocating never to remove it, 19% to sometimes remove it. 14% stating they had no opinion. While 82% advocated the evaluation of the neurological status hourly, 32% advocated the use of protocols for managing cerebral edema.

## DISCUSSION

Our survey detected that 89% of respondents were aware of a DKA protocol, a figure which is higher than that quoted by previous studies (3). This result is most likely due to the fact that the population we studied were associated with fellowship programs. With a recently reported mortality rate of 0.25% (4), pediatric DKA management will likely not change greatly in the future. However, there may be a role for clearer goals in DKA management guidelines. Providers do not agree on treatment endpoints to be used for determining resolution of DKA nor do they agree on levels of acceptable bicarbonate ranges. These results are probably due to the fact that the proposed treatment endpoints are not “normal” values but rather represent “reasonable” goals of treatment and mark a point at which treatment can be transitioned from intravenous to subcutaneous insulin. There is also a lack of consensus on insulin pump management during DKA which current guidelines (1,2) do not address. There are also inconsistencies in DKA care, such as hourly neurological monitoring stated by nearly all who took the survey, yet few have protocols for managing cerebral edema once detected. Our study suggests that there is significant discordance between current existing guidelines and treatment delivered in PEM and PCC settings, indicating a need to provide specific areas of focus for future guideline revisions.
